# Influence of early colorectal cancer component on the positive margins after endoscopic resection: a retrospective study

**DOI:** 10.1186/s12885-021-09159-8

**Published:** 2022-01-29

**Authors:** Qing-Wei Zhang, Zi-Hao Dai, Xiao-Yi Wang, Yun-Jie Gao, Zhi-Zheng Ge, Xiao-Bo Li

**Affiliations:** Division of Gastroenterology and Hepatology, Key Laboratory of Gastroenterology and Hepatology, Ministry of Health, Renji Hospital, School of Medicine, Shanghai Jiao Tong University, Shanghai Institute of Digestive Disease, Shanghai, China

**Keywords:** Early colorectal cancer, Endoscopic submucosal dissection, Endoscopic mucosal resection, Hot snare polypectomy, Vertical margin

## Abstract

**Background:**

Endoscopic treatment methods for early colorectal cancer (ECRC) mainly depend on the size and morphology. It is unclear whether different endoscopic resection methods could achieve curative resection for ECRC confined in the mucosa. The study was designed to compare the rate of positive vertical margin (VM) of ECRC with advanced adenomas (AAs) including adenoma > 1 cm, villous adenoma, high-grade intraepithelial neoplasia/dysplasia stratified by different endoscopic resection methods.

**Methods:**

Rate of positive VM for 489 ECRCs including Intramucosal (pTis) and superficial submucosal invasion (pT1) carcinomas were compared with those of 753 AAs stratified by different endoscopic resection methods using Chi-squared test. Multivariate logistic model was performed to investigate the risk factors of positive VM for different endoscopic resection methods.

**Results:**

The pTis ECRC exhibited a similar rate of positive VM as that of AAs for *en bloc* hot snare polypectomy (HSP, 0% Vs. 0.85%, *P* = 0.617), endoscopic mucosal resection (EMR, 0.81% vs. 0.25%, *P* = 0.375) and endoscopic submucosal dissection (ESD, 1.82% Vs. 1.02%, *P* = 0.659). The pTis carcinoma was not found to be a risk factor for positive VM by en bloc EMR (*P* = 0.349) or ESD (*P* = 0.368). The *en bloc* resection achieved for pT1a carcinomas exhibited similar to positive VM achieved through ESD (2.06% Vs. 1.02%, *P* = 1.000) for AAs. Nonetheless, EMR resulted in higher risk of positive VM (5.41% Vs. 0.25%, *P* < 0.001) for pT1a carcinomas as compared to AAs. The pT1a invasion was identified as a risk factor for positive VM in polyps with *en bloc* EMR (odds ratio = 23.90, *P* = 0.005) but not ESD (OR = 2.96, *P* = 0.396).

**Conclusion:**

Collectively, the pTis carcinoma was not found to be a risk factor for positive VM resected by *en bloc* HSP, EMR or ESD. Additionally, ESD may be preferred over EMR for pT1a carcinomas with lower rate of positive VM.

## Background

Early colorectal cancer (ECRC) is defined as colorectal cancer (CRC) which has not invaded beyond submucosa regardless of lymph node metastasis (LNM). With increasing implementation of national CRC screening programs worldwide, more and more ECRCs are being diagnosed [1, 2] and endoscopically resected [3, 4]. Owing to lower mortality and morbidity, endoscopic resection (ER), including polypectomy, endoscopic mucosal resection (EMR), endoscopic submucosal dissection (ESD), are more attractive to patients with ECRC as compared to surgery [5].

According to the European [6], US [7] and Japanese [8] guidelines for the management of colorectal cancers, endoscopic modalities, including polypectomy, EMR and ESD, are all eligible for resection of polyps. Even with pT1 staging, there is guarantee of complete resection and histologically confirmed low risk of lymph node metastasis (LNM). Complete resection is defined as *en bloc* the resection with negative horizontal margin (HM) and vertical margin (VM) for colorectal polyps. While positive HM is determined, endoscopic surveillance needs to be performed for 6 months owing to increased risk of local recurrence [8]. Furthermore, in patients with vertical noncurative resection, additional surgery should be done [8, 9].

According to the Japanese guideline, the choice of endoscopic modalities for colorectal cancers is based on the information of different parameters which include the size, morphology of the tumor and predicted depth of invasion [8, 10]. Although most of the endoscopic modalities are chosen depending on the first two parameters mentioned above, only a few studies have investigated whether the depth of ECRC invasion is the risk factor for positive margins of lesions resected by *en bloc* ER.

Given this background, the present study was designed to evaluate whether intramucosal ECRC or submucosal invasion in polyps contribute to higher risk of positive HM or VM as compared with advanced adenomas (AAs), identified as precancerous lesions, by different *en bloc* ER methods.

## Methods

### Patients

In this study, patients with histologically confirmed adenomas with more extensive villous architecture, high-grade intraepithelial neoplasia/dysplasia, well or moderately differentiated adenocarcinoma with intramucosal (pTis) or submucosal (pT1) staging undergoing ER in the Renji Hospital from June 2008 to June 2019 were included. The exclusion criteria followed for the patients include: 1) Final pathology of lesion was diagnosed as squamous cell carcinoma or carcinoid tumor; 2) patients had familial adenomatous polyposis; 3) patients were diagnosed with inflammatory bowel disease; 4) patients had synchronous advanced CRCs.

The primary outcomes were rates of positive VM and positive HM by different *en bloc* endoscopic resection methods and the relevant risk factors.

Informed written consent was sought from the patients before the ER of colorectal polyps. This study was approved by the institutional review board (July 2019) at the Renji Hospital. The study protocol conforms to the ethical guidelines of the 1975 Declaration of Helsinki as reflected in a priori approval by the institution’s human research committee.

### Variable and histopathological evaluation

The endoscopic resection methods used in the present included HSP, EMR and ESD. The colorectum was sub-divided into the proximal colon (cecum, ascending colon and transverse colon), the distal colon (descending colon and sigmoid) and the rectum (rectosigmoid).

All the specimens were sectioned serially at 2–3 mm intervals and subsequently stained with hematoxylin-eosin. The sections were then examined by experienced pathologists. The tumor size, histological type, HM, VM, invasion depth and lymphovascular invasion (LVI) was determined for each specimen. The tumor size was defined as the largest diameter of the lesion. Histological diagnoses was based on the World Health Organization criteria [11] and Japanese Research Society for Cancer of the Colon and Rectum (JSCCR) classification [12]. The tumors were classified as advanced adenomas (AAs) and adenocarcinoma. In this study, AAs were defined as adenomas > 1 cm, with more extensive villous architecture, or high-grade intraepithelial neoplasia/dysplasia. Adenocarcinoma was then classified as pTis or pT1 staging. Depth of the submucosal invasion was measured as per the JSCCR guidelines [8]. The tumors with pT1 staging were sub-classified as pT1a (less than 1000 μm from the muscularis mucosae) and pT1b (1000 μm more from muscularis mucosae). When the vertical margin of resected specimen was positive, the invasion depth was re-evaluated by histological examination of the specimen from additional surgery. Positive LVI was defined as the presence of cancer cells within the endothelial-line channels [13] and the *En bloc* resection was defined as a complete, single resection of the entire lesion.

### Statistical analysis

For descriptive statistics, the absolute number with proportion for categorical variable, mean and standard deviation (SD) or median and interquartile range (IQR) for continuous variable were used, respectively. The chi-square test for categorical variable, Student’s t-test or nonparametric Kruskal-Wallis rank sum test for continuous variable were used for comparisons among different patient groups, respectively. Multivariate logistic regression was performed using multivariate logistic regression model to identify the independent risk factor for outcomes of interest. Statistical analyses were performed by using R software package (version R-3.5.0, the R Foundation for statistical computing). A *P* value < 0.05 was taken as the measure of statistically significant difference.

## Results

### Clinical characteristics of the patients and lesions

A total of 1202 colorectal polyps from 1109 patients were included in the present study, including 753 (60.63%) AAs and 489 (39.37%) ECRCs. The number of polyps for HSP, EMR and ESD were 167 (13.45%), 735 (59.18%) and 340 (27.38%), respectively (Table 1).

**Table 1:** Clinical characteristics of the colorectal polyps as per different endoscopic resection methods

Characteristics	All	HSP	EMR	ESD	*P*
	1202 (100%)	167 (13.89%)	712 (59.23%)	323 (26.87%)	
**Age, year (Median, IQR)**	63(56,68)	62(57,67)	63(56,68)	64(57,69)	0.211
**Sex**					0.003
Female	437(36.36%)	62(37.13%)	233(32.72%)	142(43.96%)	
Male	765(63.64%)	105(62.87%)	479(67.28%)	181(56.04%)	
**Morphology**					< 0.001
Sessile	474(39.43)	39(23.35)	215(30.2)	220(68.11)	
Pedunculated	728(60.57)	128(76.65)	497(69.8)	103(31.89)	
**Location**					< 0.001
Rectum	347(28.87)	48(28.74)	195(27.39)	104(32.2)	
Distal colon	594(49.42)	82(49.1)	392(55.06)	120(37.15)	
Proximal colon	261(21.71)	37(22.16)	125(17.56)	99(30.65)	
**Size (cm)**					< 0.001
≤ 1 cm	355(29.53)	96(57.49)	245(34.41)	14(4.33)	
≤ 2 cm	498(41.43)	57(34.13)	366(51.4)	75(23.22)	
> 2 cm	349(29.03)	14(8.38)	101(14.19)	234(72.45)	
**Histology and invasion depth**					0.015
AA	753(62.65)	124(74.25)	428(60.11)	201(62.23)	
pTis	238(19.8)	29(17.37)	148(20.79)	61(18.89)	
pT1a	131(10.9)	6(3.59)	86(12.08)	39(12.07)	
pT1b	80(6.66)	8(4.79)	50(7.02)	22(6.81)	
**LVI**					0.455
No	1171(97.42)	165(98.8)	693(97.33)	313(96.9)	
Yes	31(2.58)	2(1.2)	19(2.67)	10(3.1)	
***En bloc*** **resection**					0.012
Yes	1099(91.43)	159(95.21)	637(89.47)	303(93.81)	
No	103(8.57)	8(4.79)	75(10.53)	20(6.19)	
**Positive HM**					0.682
No	1167(97.09)	164(98.2)	689(96.77)	314(97.21)	
Yes	35(2.91)	3(1.8)	23(3.23)	9(2.79)	
**Positive VM**					0.582
No	1156(96.17)	163(97.6)	683(95.93)	310(95.98)	
Yes	46(3.83)	4(2.4)	29(4.07)	13(4.02)	

*HSP* Hot snare polypectomy, *EMR* Endoscopic mucosal resection, *ESD* Endoscopic submucosal dissection, *IQR* Interquartile range, *AA* Advanced adenoma, *ECRC* Early colorectal cancer, *pTis* Intramucosal, *pT1a* Submucosal invasion with less than 1000 um from the muscularis mucosae, *pT1b* Submucosal invasion with 1000 um more from muscularis mucosae, *LVI* Lymphovascular invasion, *HM* Horizontal margin, *VM* Vertical margin

With respect to the rates of *en bloc* resection, HSP (91.34%) and ESD (93.81%) were higher than EMR (89.47%). According to the histological type, polyps from EMR (307, 41.77%) or ESD (139, 40.88%) had higher percentage of ECRC as compared to polyps from HSP (43, 25.75%). Additionally, the percentage of pT1 ECRC in polyps from EMR (159, 21.63%) or ESD (78, 22.94%) was also higher as compared to polyps from HSP (14, 8.38%). No significant difference was observed for LVI status, positive HM, positive VM among different endoscopic resection methods. The colorectal polyps resected by ESD tended to be sessile and larger. The detailed clinicopathological characteristics are shown in the Table 1.

### Positive margins for polyps with different histology undergoing *en bloc* HSP resection

For polyps undergoing *en bloc* HSP (Table 2), ECRC and AAs exhibited similar positive VM (2.38% Vs. 0.85%, *P* = 0.464) and positive HM (2.38% Vs. 0.85%, P = 0.464). In the multivariate logistic model with inclusion of potential risk factors for positive VM and HM, including morphology, location, size, invasion depth and LVI, pT1b invasion (OR = 14.88, 0.64–343.43, *P* = 0.092) may have insignificant influence on both of the results.

**Table 2:** Comparison of short outcomes for lesions subjected to *en bloc* HSP, EMR or ESD

	Number of lesions			Positive VM			Positive HM		
	HSP	EMR	ESD	HSP	EMR	ESD	HSP	EMR	ESD
Overall	159	637	303	2(1.26%)	14(2.20%)	7(2.31%)	2(1.26%)	11(1.73%)	7(2.31%)
AA	117	401	197	1(0.85%)	1(0.25%)	2(1.02%)	1(0.85%)	5(1.25%)	4(2.03%)
ECRC	42	236	106	1(2.38%)	13(5.51%)	5(4.72%)	1(2.38%)	6(2.54%)	3(2.83%)
pTis	29	123	55	0(0%)	1(0.81%)	1(1.81%)	0(0%)	1(0.81%)	1(1.81%)
pT1	13	113	51	1(7.69%)	12(10.62%)	4(7.84%)	1(7.69%)	5(4.42%)	2(3.92%)
pT1a	6	74	35	0(0%)	4(5.41%)	1(2.86%)	0(0%)	3(4.05%)	1(2.86%)
pT1b	7	39	16	1(14.29%)	8(20.51%)	3(18.75%)	1(14.29%)	2(5.13%)	1(6.25%)

*HSP* Hot snare polypectomy, *EMR* Endoscopic mucosal resection, *ESD* Endoscopic submucosal dissection, *AA* Advanced adenoma, *ECRC* Early colorectal cancer, *pTis* Intramucosal, *pT1a* Submucosal invasion with less than 1000 um from the muscularis mucosae, *pT1b* Submucosal invasion with 1000 um more from muscularis mucosae, *HM* Horizontal margin, *VM* Vertical margin

### Positive margins for polyps with different histology undergoing *en bloc* EMR resection

Concerning polyps *en bloc* resected by EMR (Table 2), ECRC components in polyps showed higher risk of positive VM (5.51% Vs. 0.25%, *P* < 0.001) but not HM (2.54% Vs. 1.25%, *P* = 0.345) as compared to AAs. However, it was found that pT1 ECRCs (10.62%, *P* < 0.001) including pT1a (5.41%, *P* < 0.001) and pT1b (20.51%, *P* < 0.001) but not pTis ECRCs (0.81%, *P* = 0.416) exhibited higher risk of positive VM for lesions as compared to AAs (Table 2). These findings were also validated by the multivariate logistic models. The rectum location (OR = 5.62, 1.19–26.51, *P* = 0.029), ECRC pT1a invasion (OR = 23.90, 2.55–223.76, *P* = 0.005), pT1b invasion (OR = 111.84, 1.96–1045.55, *P* < 0.001) compared to AAs were independent risk factors for positive VM (Table 3). However, none of the risk factors were found for positive HM resection (Table 4).

**Table 3:** The multivariate logistic regression analysis of risk factors for positive VM *en bloc* resected by HSP, EMR or ESD

	Positive VM								
	HSP			EMR			ESD		
	OR	95%CI	*P*	OR	95%CI	*P*	OR	95%CI	*P*
**Size**									
**≤** 1 cm	Ref			Ref			Ref		
**≤** 2 cm	2.12	0.04–125.82	0.719	0.40	0.10–1.56	0.185	0.68	–	1.000
> 2 cm	0.00	–	0.998	0.43	0.06–3.08	0.401	0.00	–	0.998
**Morphology**									
Sessile	Ref			Ref			Ref		
Pedunculated	0.16	0.00–7.89	0.36	0.79	0.21–3.01	0.732	0.26	0.02–3.47	0.307
**Location**									
Proximal colon	Ref			Ref			Ref		
Distal colon	0.00	–	0.997	1.4	0.13–15.40	0.785	10.84	0.46–253.85	0.139
Rectum	2.10	0.08–55.64	0.656	5.62	1.19–26.51	0.029	2.67	0.18–39.06	0.473
**Histology and invasion depth**									
AA	Ref			Ref			Ref		
ECRC pTis	0.00	–	0.997	3.99	0.24–65.83	0.333	3.18	0.26–39.60	0.369
ECRC pT1a	0.00	–	0.999	23.90	2.55–223.76	0.005	2.96	0.24–36.43	0.396
ECRC pT1b	14.88	0.64–343.43	0.092	111.84	1.96–1045.55	< 0.001	142.87	7.82–2803.00	0.001
**LVI**									
negative	Ref			Ref			Ref		
positive	0.67	–	1.000	3.66	0.56–23.89	0.175	1.12	0.05–23.52	0.942

*VM* Vertical margin, *HSP* Hot snare polypectomy, *EMR* Endoscopic mucosal resection, *ESD* Endoscopic submucosal dissection, *AA* Advanced adenoma; ECRC: early colorectal cancer, *pTis* Intramucosal, *pT1a* Submucosal invasion with less than 1000 um from the muscularis mucosae, *pT1b* Submucosal invasion with 1000 um more from muscularis mucosae, *LVI* Lympovascular invasion

**Table 4:** The multivariate logistic regression analysis of risk factors for positive HM *en bloc* resected by HSP, EMR or ESD

	Positive HM								
	HSP			EMR			ESD		
	OR	95%CI	*P*	OR	95%CI	*P*	OR	95%CI	*P*
**Size**									
≤ 1 cm	Ref			Ref			Ref		
≤ 2 cm	2.12	0.04–125.82	0.719	1.27	0.34–4.77	0.719	0.52	–	1.000
> 2 cm	0.00	–	0.998	0.00	–	0.993	0.00	–	0.998
**Morphology**									
Sessile	Ref			Ref			Ref		
Pedunculated	0.16	0.00–7.89	0.36	0.53	0.15–1.91	0.331	0.68	0.07–6.92	0.738
**Location**									
Proximal colon	Ref			Ref			Ref		
Distal colon	0.00	–	0.997	0.00	–	0.992	0.00	–	0.994
Rectum	2.10	0.08–55.64	0.656	2.71	0.73–10.06	0.136	0.00	–	0.994
**Histology and invasion depth**									
AA	Ref			Ref			Ref		
ECRC pTis	0.00	–	0.997	0.62	0.07–5.52	0.67	1.67	0.17–16.74	0.661
ECRC pT1a	0.00	–	0.999	3.18	0.71–14.14	0.129	1.49	0.15–14.90	0.732
ECRC pT1b	14.88	0.64–343.43	0.092	3.36	0.50–22.75	0.213	35.55	1.44–879.44	0.029
**LVI**									
negative	Ref			Ref			Ref		
positive	0.67	–	1.000	2.42	0.19–30.28	0.494	0.00	–	0.998

*HM* Horizontal margin, *HSP* Hot snare polypectomy, *EMR* Endoscopic mucosal resection, *ESD* Endoscopic submucosal dissection, *AA* Advanced adenoma, *ECRC* Early colorectal cancer, *pTis* Intramucosal, *pT1a* Submucosal invasion with less than 1000 um from the muscularis mucosae, *pT1b* Submucosal invasion with 1000 um more from muscularis mucosae, *LVI* Lympovascular invasion

### Positive margins for polyps with different histology undergoing *en bloc* ESD resection

With respect to polyps undergoing *en bloc* ESD (Table 2), the ECRC components in polyps exhibited higher risk of positive VM (4.72% Vs. 1.02%, *P* = 0.041) but not positive HM (2.83% Vs. 2.03%, *P* = 0.659) to AAs. Further subgroup analyses (Table 2) revealed pT1 ECRC (7.84%, *P* = 0.005), pT1b (18.75%, *P* < 0.001) but not pT1a (2.86%, *P* = 0.374) or pTis ECRC (1.82%, *P* = 0.627) to exhibit higher risk of positive VM as compared to AAs. The multivariate logistic models only identified pT1b invasion (OR = 142.87, 7.82–2803.00, *P* = 0.001) as independent risk factors for positive VM as compared to AAs (Table 3). Moreover, multivariate logistic regression also found pT1b invasion (OR = 35.55, 1.44–879.44, *P* = 0.029) to be an independent risk factor for positive HM in polyps with *en bloc* ESD (Table 4).

## Discussion

To the best of our knowledge, present study for the first time investigates whether depth of invasion of ECRC contributes to the higher risk of positive VM or HM after different *en bloc* ER. The present study demonstrated that pTis ECRC did not contribute to risk of positive VM or HM irrespective of endoscopic resection methods. The multivariate regression model identified pT1a invasion and pT1b invasion as independent risk factors for the positive VM in patients undergoing *en bloc* EMR. When *en bloc* resection was achieved, ESD showed similar curative vertical resection for pT1a to AAs.

Usually, HSP is applied for the resection of small or pedunculated polyps [14, 15], which coincides with lesions resected by HSP in the present study. It has been reported that muscularis mucosa could be obtained as high as 96% and obtaining of submucosal tissue could be achieved as high as 81% in polyps resected by HSP [16], suggestive of applicability of HSP for resection of pTis or even pT1 carcinoma. In the present study, complete resection for all pTis carcinomas was achieved by *en bloc* HSP and comparable curative vertical and horizontal resection rate between AAs and ECRC was confirmed, though only 13 pT1 lesions were included for the analysis. A previous study has reported that CRC to be detectable in 0.46% of polyps sized **≤**5 mm in diameter and 3.3% of polyps with a diameter of 6–9 mm and that submucosal invasive cancer is as rare as 0.1% in small polyps [17]. Therefore, it may be safe to resect most of the small polyps by HSP under the premise of *en bloc* resection. However, close endoscopic observation should be carried out to suspected pT1 carcinomas in small polyps and HSP resection needs to be performed prudently since 19% of submucosal tissue for polyps by HSP could not be obtained and 14.29% (1/13) polyp was resected with positive VM and HM in the present study.

The EMR has been shown to achieve as high as 92% (105/114) of submucosa obtaining [18], which was consistent with the results of the present study that no difference in positive VM rate was observed between AAs and pTis ECRCs after *en bloc* resection. Similar results were observed in polyps with *en bloc* ESD, indicative of no influence of pTis ECRC on the positive VM irrespective of resection methods. Therefore, it may be reasonable to choose HSP, EMR and ESD based on the information available on the size and morphology of the tumor to resect pTis ECRCs [8] and there is no need to use more aggressive resection methods for polyps with pTis ECRC if *en bloc* resection could be guaranteed.

We further analyzed whether EMR was suitable for resection of pT1 ECRCs. pT1a invasion and pT1b invasion were found to be independent risk factors for positive VM with *en bloc* EMR. A previous study has reported that the median resection depth from muscularis mucosae was 338 μm in EMR resection, though 92% of submucosa could be obtained [19]. This indicated that some cases of pT1 ECRCs could not be resected for negative VM due to its limited resection depth. In the present study, 10.62% of positive VM could be observed in pT1 ECRCs with *en bloc* EMR. In contrary, as low as 2.86% of positive VM for pT1a ECRCs were achieved for ESD when *en bloc* resection was achieved. The results of multivariate logistic regression models did not identify pT1a invasion as an independent risk factor for positive VM in polyps with *en bloc* ESD. Since *en bloc* EMR not ESD resulted in higher rate of positive VM for pT1a carcinomas as compared to AAs, ESD may be preferred over EMR for pT1a carcinomas. Consistently, pT1a ECRC is an indication for ESD according to the Japan Gastroenterological Endoscopy Society (JGES) guideline [18]. However, pT1a should be carefully differentiated from pT1b and the latter should be sent for surgery. In the present study, 80 pT1b (16.95%) ECRCs were misdiagnosed as pTis or pT1a ECRCs and underwent endoscopic resection, which indicated a need for extra caution during the diagnosis of endoscopically curable ECRCs by the application of magnifying endoscopy [20].

The present study suffers from several limitations. First, although a large number of colorectal polyps were included for analysis in this study, it was a study performed in single hospital. Second, only positive HM and VM status was evaluated in this study. Systematic evaluation of recurrence and metastasis after polyp resection could not be performed because most of colorectal polyps with positive HM and VM underwent additional ER or surgery and long-term follow-up was unavailable. Further studies are required to analyze whether recurrence differs among different endoscopic methods. Third, in our retrospective study, selection criteria for HSP, EMR or ESD for colorectal polyps may differ slightly among different endoscopists. Percent of sessile polyps or polyps sized more than 2 cm also differed among polyps with different ER methods due to difficulty of *en bloc* EMR or HSP resection to sessile polyps or large polyps. This contributed to the selection bias and could not be clarified in this study. To a certain extent, analysis based on real-world clinical practice may generalize our results. And a prospective study needs to be performed to eliminate this bias. Fourth, some pathology related information such as the median resection depth, could not be collected and requires prospective study. Fifth, we were unable to investigate whether pT1a or pT1b components contributed to the higher risk of positive VM as compared to AAs in polyps undergoing HSP due to the small number of cases. Further study involving larger number of lesions is needed for investigation.

Taken together, choice of endoscopic resection methods based on size and morphology of the tumor in guarantee of *en bloc* resection for treatment of pTis carcinomas was rational. ESD was preferred over EMR for pT1a carcinomas with lower rate of positive VM.

## Data Availability

The datasets generated and/or analysed during the current study are not publicly available due personal information involved but are available from the corresponding author on reasonable request.

## Abbreviations

ECRC: Early colorectal cancer
AA: Advanced adenoma
ER: Endoscopic resection
HSP: Hot snare polypectomy
EMR: Endoscopic mucosal resection
ESD: Endoscopic submucosal dissection
VM: Vertical margin
HM: Horizontal margin
